# CASCAD: a database of annotated candidate single nucleotide polymorphisms associated with expressed sequences

**DOI:** 10.1186/1471-2164-6-10

**Published:** 2005-01-27

**Authors:** Victor Guryev, Eugene Berezikov, Edwin Cuppen

**Affiliations:** 1 Hubrecht Laboratory, Netherlands Institute for Developmental Biology, Uppsalalaan 8, 3584CT, Utrecht, The Netherlands

## Abstract

**Background:**

With the recent progress made in large-scale genome sequencing projects a vast amount of novel data is becoming available. A comparative sequence analysis, exploiting sequence information from various resources, can be used to uncover hidden information, such as genetic variation. Although there are enormous amounts of SNPs for a wide variety of organisms submitted to NCBI dbSNP and annotated in most genome assembly viewers like Ensembl and the UCSC Genome Browser, these platforms do not easily allow for extensive annotation and incorporation of experimental data supporting the polymorphism. However, such information is very important for selecting the most promising and useful candidate polymorphisms for use in experimental setups.

**Description:**

The CASCAD database is designed for presentation and query of candidate SNPs that are retrieved by *in silico *mining of high-throughput sequencing data. Currently, the database provides collections of laboratory rat (*Rattus norvegicus*) and zebrafish (*Danio rerio*) candidate SNPs. The database stores detailed information about raw data supporting the candidate, extensive annotation and links to external databases (e.g. GenBank, Ensembl, UniGene, and LocusLink), verification information, and predictions of a potential effect for non-synonymous polymorphisms in coding regions. The CASCAD website allows search based on an arbitrary combination of 27 different parameters related to characteristics like candidate SNP quality, genomic localization, and sequence data source or strain. In addition, the database can be queried with any custom nucleotide sequences of interest. The interface is crosslinked to other public databases and tightly coupled with primer design and local genome assembly interfaces in order to facilitate experimental verification of candidates.

**Conclusions:**

The CASCAD database discloses detailed information on rat and zebrafish candidate SNPs, including the raw data underlying its discovery. An advanced web-based search interface  allows universal access to the database content and allows various queries supporting many types of research utilizing single nucleotide polymorphisms.

## Background

Single nucleotide polymorphisms (SNPs) are the most common form of genetic variation within species. As a result, SNPs are now becoming the most popular type of marker in genetic association and mapping studies. SNPs are also most likely to be the molecular basis for the majority of phenotypic variation in (outbred) populations. In particular, SNPs in regulatory and protein-coding regions can have an effect on gene expression levels and protein activity, respectively. The phenotypic differences observed between selected (sub) strains in model organisms may be the result of specific (combinations of) natural occurring polymorphisms. Hence, a comprehensive inventory of SNPs, including extensive annotation will be extremely valuable in the search for functional polymorphisms.

There is often a vast unexplored potential in large sequence datasets that have been collected for other purposes, for example, EST and whole genome sequencing (WGS) projects. In an effort to address these two issues, we have developed an *in silico *candidate SNP mining pipeline that uses all publicly available sequence data for a specific organism, and designed a database, CASCAD (CAscad SNP CAndidates Database), that allows storage of a wide variety of primary source data, cross-annotation to other databases, and analysis parameters for SNPs associated with expressed sequences.

## Construction and content

We applied the SNP discovery pipeline to both rat [1] and zebrafish (unpublished results) and identified about 33,000 and 52,000 high-quality candidates, respectively, that were extensively annotated and stored in the CASCAD database (Table 1). The database includes detailed primary information on which the discovery of the candidate SNPs was based. This information, including sequence quality information (Phred score), the number of supporting reads for every nucleotide observed at the SNP position, and expected alignment lengths, was found to be very valuable for filtering for predicted variants that have the highest likelihood to be experimentally confirmed. Two verification experiments for the rat resulted in confirmation rate estimates of 59% (68 candidate SNPs in 10 different laboratory rat strains) and 50.3 % (340 candidates in 5 laboratory and 2 wild rat isolates) [1]. A set of 139 CASCAD entries tested in 7 zebrafish isolates confirmed 67.6% of them as true polymorphisms (unpublished data). The success rate values obtained are likely to be underestimates since only limited number of isolates/samples were used and we were unable to include exactly the same isolates that were used for generating the primary data (e.g. EST sequencing).

**Table 1:** Input data (number of sequence reads) for the CASCAD pipeline and number of predicted candidate SNPs.

	***Rattus norvegicus***	***Danio rerio***
Input data		
mRNA	25, 634	3, 366
EST	244, 518	283, 572
WGS	19, 813, 313	11, 588, 394

Candidate SNPs predicted	33, 305	51, 769
synonymous	3, 842	9, 111
non-synonymous	3, 708	6, 217
nonsense	162	158

**Figure 1:**
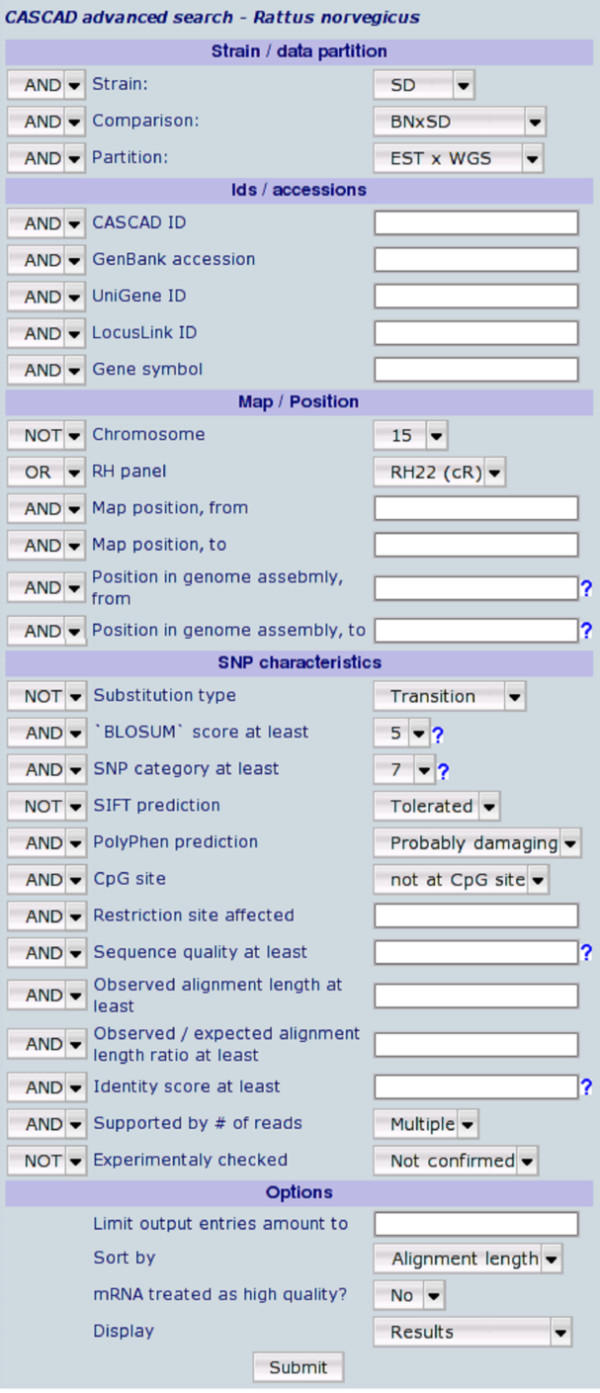
CASCAD advanced search form.

We designed a web-based interface with Perl scripts communicating to a MySQL database, and displaying HTML pages through Apache server running on SuSE Linux. The interface provides simple, advanced (Figure 1), and sequence-based search forms. Parameters that can be used in a search include strain information, different formats of sequence identifiers (e.g. GenBank, UniGene, LocusLink accessions or gene symbol), map positions (genetic and physical), and a wide variety of SNP characteristics, such as experimental evidence, a likelihood score for verification as deduced from extensive verification experiments [1], and information regarding restriction sites that have been affected, facilitating the design of RFLP based assays. To this end CASCAD is tightly linked to primer design [2] and local genome assembly [3] interfaces, enabling a fast, reliable, and universal primer design for a chosen SNP candidate even when there is no assembled genome sequence available. To facilitate the retrieval of candidate SNPs with higher verification rates, represented by more reliable and common SNP candidates, we implemented the possibility to restrict searches to entries characterized by location at hypervariable CpG site, by minimal basecalling quality (Phred score), sequence match size, and/or minimal number of supporting reads for either allele.

In addition to primary sequence data analysis, the effect of all SNPs on protein coding capacity was evaluated and non-synonymous SNPs were categorized in classes reflecting the severity of the polymorphism using a BLOSUM-based score. The predicted missense SNPs were analyzed by SIFT [4] and Polyphen [5] programs that utilize not only substitution information but also phylogenetic conservation and structural protein information to predict a potential effect of the polymorphism on protein function.

**Figure 2:**
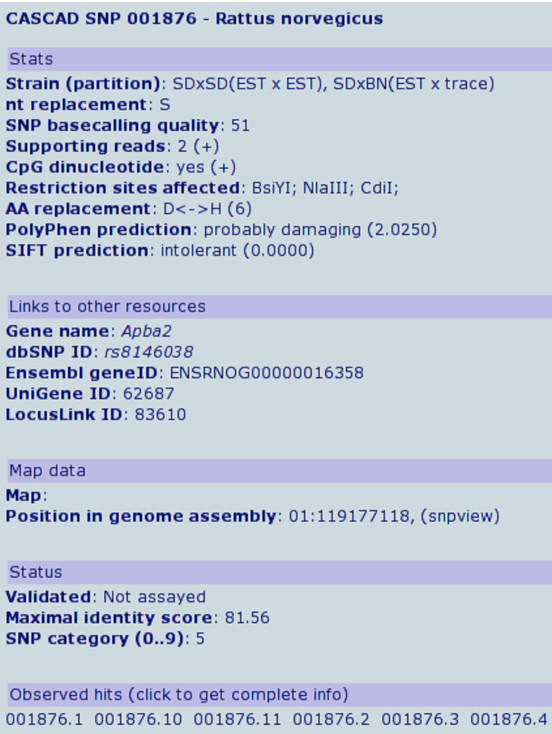
SNP details page

Query results are summarized on the SNP details page (Figure 2), listing the SNP characteristics and including active links to other databases and resources, such as dbSNP, Ensembl, UniGene, and LocusLink. More detailed information regarding raw data underlying the candidate SNP (links to the original sequence files and a full nucleotide and protein alignment) can be obtained by clicking on the observed nearly exact hits between nucleotide sequences. Moreover, statistics on the data that support the SNP (number of occurrences for every nucleotide at SNP position, range of Phred basecalling quality scores) are provided.

## Utility and discussion

For many applications, it is important to be able to distinguish between SNP candidates by their characteristics, as they may be predictive for verification success rate or carry biologically relevant information. Non-confirmed candidate polymorphisms may represent variants uncommon for a given population, but also sequencing errors (all types of sequences), RNA editing events and reverse transcriptase errors (EST reads). In order to minimize the contribution of false positives, one can exclude polymorphisms based on a single read for either allele, as is common for many *in silico *discovery pipelines [6]. Although this is a valid approach when selecting SNPs for population or association genetics, one could inadvertently discard many rare variants that may be associated with phenotypic variation, for example by affecting protein structure or function. Information on such polymorphisms can be very useful when mapping disease or QTL alleles.

We have developed our database to fulfill the needs of any particular SNP application by providing control over every parameter we used in the polymorphism discovery step.

Applications of the CASCAD database include queries for potentially deleterious SNPs in a specific genomic region of interest, for example a QTL interval, design of SNP-based mapping panels using either RFLP or any other technology, and identification of informative SNPs for fine-mapping. Custom sequences can be provided to search for known SNPs in any sequence of interest. In addition, the CASCAD pipeline [1] can be used to build a candidate SNP database for any model organism of interest for which sufficient sequencing data is available.

## Conclusions

The main purpose of CASCAD database is to provide flexible access to candidate single nucleotide polymorphisms, which were predicted using a computational approach from publicly available sequence data of the rat and zebrafish. The resulting database is crosslinked to most common public databases and can be queried for SNPs using accession numbers, sequence context, SNP characteristics, but also using parameters specific to the SNP discovery process, allowing stringent or relaxed conditions suitable for different types of applications.

## Availability and requirements

The database is freely accessible through the website . Programs, scripts, MySQL database dumps, and instructions for setting up a species-specific SNP database can be obtained from the authors upon request.

## Authors' contributions

VG designed and implemented the CASCAD database. EB tested database and interface. EC provided supervision and guidance for the project.
